# The Doubletime Homolog *KIN-20* Mainly Regulates *let-7* Independently of Its Effects on the Period Homolog *LIN-42* in *Caenorhabditis elegans*

**DOI:** 10.1534/g3.118.200392

**Published:** 2018-06-07

**Authors:** Kyle Rhodehouse, Katherine Cascino, Laura Aseltine, Allegra Padula, Rachel Weinstein, Joseph S. Spina, Christiane E. Olivero, Priscilla M. Van Wynsberghe

**Affiliations:** Department of Biology, Colgate University, Hamilton, NY 13346

**Keywords:** *KIN-20*, *LIN-42*, *let-7*, miRNA

## Abstract

The *Caenorhabditis elegans* (*C. elegans*) heterochronic pathway, which regulates developmental timing, is thought to be an ancestral form of the circadian clock in other organisms. An essential member of this clock is the Period protein whose homolog, *lin-42*, in *C. elegans* is an important heterochronic gene. **LIN-42** functions as a transcriptional repressor of multiple genes including the conserved **lin-4** and *let-7* microRNAs. Like other Period proteins, levels of *LIN-42* oscillate throughout development. In other organisms this cycling is controlled in part by phosphorylation. *KIN-20* is the *C. elegans* homolog of the *Drosophila* Period protein kinase Doubletime. Worms containing a large deletion in *kin-20* have a significantly smaller brood size and develop slower than wild type *C. elegans*. Here we analyze the effect of *kin-20* on *lin-42* phenotypes and microRNA expression. We find that *kin-20* RNAi enhances loss-of-function *lin-42* mutant phenotypes and that *kin-20* mutant worms express lower levels of *LIN-42*. We also show that *kin-20* is important for post-transcriptional regulation of mature *let-7* and *lin-4* microRNA expression. In addition, the increased level of *let-7* found in *lin-42(n1089)* mutant worms is not maintained after *kin-20* RNAi treatment. Instead, *let-7* is further repressed when levels of *kin-20* and *lin-42* are both decreased. Altogether these results suggest that though *kin-20* regulates *lin-42* and *let-7* microRNA, it mainly affects *let-7* microRNA expression independently of *lin-42*. These findings further our understanding of the mechanisms by which these conserved circadian rhythmic genes interact to ultimately regulate rhythmic processes, developmental timing and microRNA biogenesis in *C. elegans*.

Many organisms exhibit circadian rhythmic behaviors that cycle in accordance with the Earth’s rotation and thus exhibit periodicities of ∼24 hr. These circadian rhythms can be entrained by environmental stimuli and persist in the absence of such cues, are unchanged by variations in temperature, and can be reset by external stimuli. Other fundamental aspects of life are governed by ultradian or infradian rhythms with periods that are either less or greater than the canonical 24 hr circadian rhythm, respectively. Central to these rhythms is the circadian clock, a group of highly conserved genes that function in oscillating feedback loops that are controlled by both transcriptional and post-transcriptional mechanisms.

In the nematode *C. elegans*, locomotion, resistance to osmotic stress, melatonin biosynthesis, and multiple metabolic variables can be entrained by light cues with a daily periodicity (Kippert *et al.* 2002; Saigusa *et al.* 2002; Migliori *et al.* 2011, 2012). Other rhythmic behaviors in *C. elegans*, like olfactory response, have been entrained by alterations in temperature (Olmedo *et al.* 2012). *C. elegans* also exhibits other essential ultradian rhythms like defecation (Iwasaki *et al.* 1995; Kobayashi *et al.* 2011). Though *C. elegans* do not express many of the proteins found in the classical circadian clocks of mammals and *Drosophila*, they do express many core clock proteins (Banerjee *et al.* 2005; Romanowski *et al.* 2014). Interestingly, many of these core clock proteins also function in the heterochronic pathway of *C. elegans* that regulates developmental timing (Banerjee *et al.* 2005; Temmerman *et al.* 2011). Thus the heterochronic pathway has been hypothesized to be an ancestral form of the circadian clock system (Temmerman *et al.* 2011).

Like the core circadian clock, the heterochronic pathway consists of many genes that successively regulate one another through feedback mechanisms to ultimately promote *C. elegans* development through four larval stages and into adulthood (Rougvie and Moss 2013). Proper progression through the heterochronic pathway can be assessed by evaluating the development of heterochronic seam cells (Resnick *et al.* 2010). Specific numbers of seam cells are present during each larval stage, and after the L4-to-adult molt, seam cells exit the cell cycle, fuse and generate a ridged cuticle structure called alae (Resnick *et al.* 2010). Thus, heterochronic mutants often display altered seam cell numbers and/or precocious or retarded alae formation (Resnick *et al.* 2010).

Based on its sequence similarity and rhythmic expression, the heterochronic gene *lin-42* is the *C. elegans* homolog of the Period protein (Jeon *et al.* 1999). Period proteins act as transcriptional repressors to regulate circadian rhythms (Hardin 2005; Yu and Hardin 2006). *LIN-42* also acts as a transcription factor to regulate expression of a multitude of genes (Mcculloch and Rougvie 2014; Van Wynsberghe and Pasquinelli 2014; Van Wynsberghe *et al.* 2014; Perales *et al.* 2014). In addition, *lin-42* is essential for proper developmental timing, molting, and entry into an alternative dauer larval stage (Tennessen *et al.* 2006, 2010; Monsalve *et al.* 2011; Edelman *et al.* 2016). Consequently, *lin-42* mutant worms show defects in circadian rhythmic activity, and exhibit a dumpy phenotype and precocious alae formation (Abrahante *et al.* 1998; Simonetta *et al.* 2009; Van Wynsberghe and Pasquinelli 2014).

Oscillation of Period is controlled both transcriptionally and post-transcriptionally. One protein important for this regulation is the kinase Doubletime in *Drosophila* and Casein Kinase 1ε and δ (CKIε/δ) in mammals (Kloss *et al.* 1998; Price *et al.* 1998). Phosphorylation by Doubletime decreases the stability and thus the levels and subcellular localization of Period (Price *et al.* 1998; Cyran 2005; Crane and Young 2014). The *C. elegans* homolog of Doubletime and CK1ε/δ is *KIN-20* (Banerjee *et al.* 2005). RNAi against *kin-20* causes some precocious developmental timing defects (Banerjee *et al.* 2005), though the reason for these defects and the effect of *KIN-20* on the Period protein homolog *LIN-42* is unknown.

Some of the genes associated with the heterochronic pathway function as small RNAs called microRNAs (miRNAs) (Rougvie and Moss 2013). miRNAs post-transcriptionally regulate gene expression by binding imperfectly to target mRNAs to ultimately inhibit their expression (Finnegan and Pasquinelli 2013). *LIN-42* negatively regulates a broad range of miRNAs including *let-7* and *lin-4* (Mcculloch and Rougvie 2014; Van Wynsberghe *et al.* 2014; Perales *et al.* 2014). The conserved miRNA *let-7* is essential for promoting cellular differentiation later in development (Resnick *et al.* 2010; Mondol and Pasquinelli 2012; Lee *et al.* 2016). Accordingly, under-expression of *let-7* is associated with retarded development and a bursting vulva phenotype in *C. elegans*, as well as breast, lung and colon cancer in humans (Sayed and Abdellatif 2011; Mondol and Pasquinelli 2012; Lee *et al.* 2016). Loss of the conserved *lin-4* microRNA, which is first expressed during the first larval stage, also causes retarded development in *C. elegans* (Ambros and Horvitz 1984; Ambros 1989).

miRNAs like *let-7* and *lin-4* ultimately function as ∼22 nucleotide (nt) RNAs, however they are initially transcribed from the genome by RNA polymerase II into long, primary miRNAs (pri-miRNAs) that are subsequently capped and polyadenylated (Lee *et al.* 2004). These primary miRNAs are then processed by the Microprocessor complex, which is composed of the RNase III enzyme Drosha and the RNA binding protein DGCR8 (also known as Pasha), into a ∼70 nt hairpin structured precursor miRNA (pre-miRNA) (Resnick *et al.* 2010; Finnegan and Pasquinelli 2013). Following export to the cytoplasm, the pre-miRNA is further processed by a second RNase III enzyme Dicer into the ∼22 nt mature miRNA (Resnick *et al.* 2010; Finnegan and Pasquinelli 2013). This mature miRNA is loaded onto Argonaute to form the miRNA-induced silencing complex (miRISC) (Resnick *et al.* 2010; Finnegan and Pasquinelli 2013). miRISC then uses the miRNA as a guide to bind and downregulate target gene expression (Resnick *et al.* 2010; Finnegan and Pasquinelli 2013). Consequently, aberrant levels of the mature miRNA can cause inappropriate expression of target genes and thus subsequent phenotypic effects. To ensure proper miRNA expression, each step in miRNA biogenesis is subject to regulation (Finnegan and Pasquinelli 2013). Some regulators act on a specific miRNA, while others act more globally to control miRNA expression (Finnegan and Pasquinelli 2013; Lee *et al.* 2016).

Here we utilized a large deletion of *kin-20*, *kin-20(ok505)*, to analyze the effect of *kin-20* on organismal development, *lin-42* phenotypes and expression, and *let-7* and *lin-4* miRNA biogenesis. We found that *kin-20* mutants have significantly fewer progeny and a slower growth than WT N2 worms, though they do not exhibit classical developmental timing defects in alae production. Because *kin-20(ok505);lin-42(n1089)* worms were lethal, we analyzed the impact of *kin-20* RNAi on *lin-42(n1089)* mutant worms. We found that under-expression of *kin-20* enhanced *lin-42(n1089)* mutant phenotypes including precocious alae formation. Consistent with these phenotypic results, we found that LIN-42A levels were decreased in *kin-20(ok505)* mutant worms. Like *LIN-42*, *KIN-20* is important for both *let-7* and *lin-4* miRNA expression. Though it is possible the decrease in *let-7* levels in *kin-20* mutant worms is dependent on LIN-42A, our results more strongly suggest that *KIN-20* impacts expression of the *let-7* miRNA independently of *LIN-42*, since *KIN-20* had no effect on a third constitutively-expressed, non-heterochronic miRNA target miR-58.1, and *KIN-20* did not impact primary *let-7* transcription. In addition, growth of *lin-42(n1089)* mutant worms on *kin-20* RNAi caused a further reduction in *let-7* levels. Altogether these results suggest that although *KIN-20* regulates both *LIN-42* and some miRNAs in *C. elegans*, *KIN-20* mainly does so in a manner unpredicted by the inhibitory function of Doubletime (the *KIN-20* homolog) on Period (the *LIN-42* homolog) in *Drosophila*.

## Materials and Methods

### Nematode strains and culture conditions

The following *C. elegans* strains were used: wild type (WT) N2 Bristol, *kin-20(ok505)* (VC398), *lin-42(ok2385)* (RB1843), and *lin-42(n1089)* (MT2257). The integrated transgene wls79 contains ajm-1::gfp/MH27:: GFP and scm-1::GFP to allow visualization of seam cells. We crossed animals containing wls79 to VC398 to generate wls79;*kin-20*(ok505) worms. The integrated strain PQ462 contains 1568 bp of *let-7* promoter sequence driving nuclear-localized GFP expression (p**let-7**B::GFP) (Kai *et al.* 2013). We crossed PQ462 to VC398 to generate p**let-7**B::GFP;*kin-20(ok505)*.

Worms were maintained at 15° or 20° and synchronized by standard hypochlorite treatment. Starvation-arrested L1 worms were plated on *E. coli* OP50-seeded plates at 25° and collected at the appropriate time point. Larval stages correspond to the timing of development for WT N2 worms based on previously published time course analyses of worm development and molting at 25° (Jeon *et al.* 1999; Zisoulis *et al.* 2012), as *lin-42* mutants develop somewhat asynchronously (Monsalve *et al.* 2011). We performed two-generation feeding RNAi experiments as described (Bracht *et al.* 2010) except that the IPTG concentration was increased to 10 mM. Briefly, L1 stage worms were grown on RNAi plates at 15°. Then synchronized, starved L1 progeny from these worms were grown on the same RNAi food at 25° until the desired stage before molecular or phenotypic analysis.

Brood counts were analyzed of synchronized, singled WT N2 or *kin-20(ok505)* worms grown at 15° or 25°. Parental worms were passaged to new plates over the course of the experiment to enable detection of all progeny. Death and dumpy phenotypes were analyzed in at least 500 adult animals grown on RNAi for two-generations as described above. The presence or absence of adult alae on the cuticle was measured in at least 20 synchronized animals after growth at 25° until the L4 or young adult stage. Alae was classified as complete if it extended continuously over all seam cells in three perfectly parallel ridges. Animals that had alae that was not complete and/or did not contain three perfectly parallel ridges were classified as having abnormal alae. Seam cell nuclei were counted in at least 19 synchronized animals grown at 25° until the L4 or young adult stage. Fluorescent microscopy analysis was performed on at least 25 synchronized animals after growth at 25° until the L3 or L4 stage. Fluorescent micrographs were captured under equivalent exposure times.

Statistical differences of sample phenotypes were analyzed as appropriate by Student’s *t*-tests or chi-square tests.

### RNA analyses

Total RNA was extracted from synchronized, staged worm populations using TRIzol reagent (Life Technologies), and cDNA synthesis was completed with TaqMan microRNA assays (Thermo Fisher Scientific) or as previously described with random oligos or oligo dT (Van Wynsberghe *et al.* 2011a). qPCR was performed with TaqMan or SYBR Green reagents (Thermo Fisher Scientific) and 6.25 pmol of each primer (Table S1) on an ABI Prism 7900 or a Thermo Fisher Scientific QuantStudio 3 Real-time PCR system. Statistical differences of RNA levels between samples were analyzed by Student’s *t*-tests or two-way ANOVAs.

### Protein analyses

Polyclonal antibodies against the C terminal sequence (KTSSSSSLLMLRDSQNE) of *LIN-42* were raised in rabbits and purified by YenZym Antibodies, LLC. Western blotting was performed as described with this rabbit polyclonal antibody against *LIN-42* (YenZym Antibodies, LLC) or a mouse monoclonal antibody against tubulin (Sigma-Aldrich) (Van Wynsberghe *et al.* 2011b). The HRP conjugated goat anti-rabbit or mouse secondary antibodies (ThermoFisher Scientific) were used and visualized on a ChemiDoc XRS+ (BioRad) system.

### Data Availability

Reagents will be made available upon request. The authors state that all data necessary for confirming the conclusions presented in the article are represented fully within the article. Supplemental material available at Figshare: https://doi.org/10.25387/g3.6225893.

## Results

**Figure 1:**
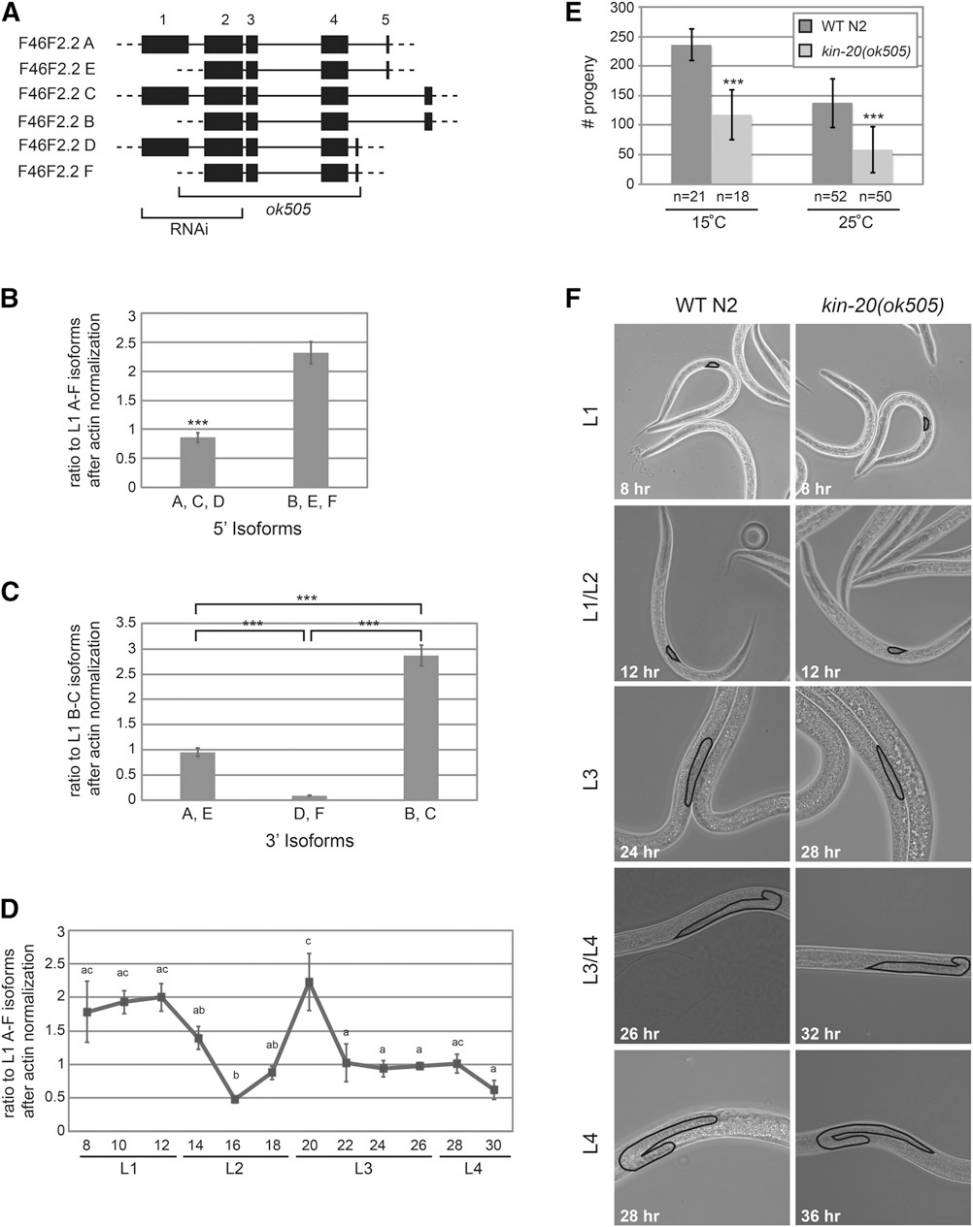
*KIN-20* is required for proper developmental timing. (A) Depiction of the *kin-20* gene. According to WormBase there are 6 isoforms, A-F, each with alternative 5′ and 3′ UTRs. Each of the 6 isoforms is paired such that exons 2-5 are identical in each member of the pair. In addition, isoforms A, C and D contain exon 1, while isoforms E, B and F start at the 2^nd^ in frame AUG start codon at nt 6 of exon 2. Untranslated sequences are depicted by dashed lines, and introns are depicted by solid lines. Regions impacted by RNAi or the *ok505* allele are shown. (B-D) Synchronized, starved L1 WT N2 worms were grown at 25°C and collected every 2 hr from 8 to 30 hr after plating on food. RNA was extracted, reverse transcribed into cDNA, and analyzed by qPCR with primers located in exons 1 and 2 (to amplify isoforms A, C and D), in exons 2 and 3 (to amplify all isoforms A-F), or in exon 4 and each unique exon 5 (to amplify the distinct 3′ isoforms) of *KIN-20*. Data are shown from 3 independent experiments and was analyzed by a two-way ANOVA (***, *P* < 0.0005). Error bars show s.e.m. (B) The average ratios of the B, E and F isoforms and the A, C, and D isoforms are shown after normalization to the L1 A-F isoforms and actin. (C) The average ratios of the A, E isoforms, the D, F isoforms and the B, C isoforms are shown after normalization to the L1 B-C isoforms and actin. (D) The ratio of all isoforms of KIN20 after normalization to actin is shown. Letters indicate time points that are not significantly different from one another. More specifically, all time points marked “ac” are not significantly different from time points marked with an “a” or a “c”, but are significantly different from time points marked with only a “b”. (E) The average progeny number of *kin-20(ok505)* mutant worms compared to WT N2 worms was calculated from the indicated number of worms grown at 15°C and 25°C and analyzed by Student’s *t*-tests (***, *P* < 0.0005). Error bars show s.e.m. (F) Synchronized WT N2 worms were grown at 25°C until the times shown. Synchronized *kin-20(ok505)* worms were also grown at 25°C until the same stage in development as WT N2, as determined by gonad size. Representative images were taken at 400x magnification. Gonads are outlined.

*KIN-20* is a homolog of the Period protein kinase Doubletime in *Drosophila* and casein kinase I ε/δ in mammals (Banerjee *et al.* 2005). Like other circadian clock homologs in *C. elegans*, *kin-20* is important for proper developmental timing (Banerjee *et al.* 2005). Previous research has utilized RNAi to ascertain the impact of *kin-20* on developmental timing (Banerjee *et al.* 2005). To more completely abrogate *kin-20* expression, we utilized the *ok505* allele that causes an approximately 2.2 kb deletion in the *kin-20* transcript that removes almost the entire *kin-20* gene (Figure 1A). According to WormBase, the *kin-20* gene encodes 6 distinct isoforms (labeled A-F) that each have different 5′ and 3′ UTRs of varying lengths and splice patterns. However, the internal sequence (exons 2-4) of all isoforms is shared (Figure 1A). In its simplest form there are 3 isoform pairs (A/E, C/B, and D/F) that share exons 2-5 (Figure 1A). In addition, one member of each pair (isoforms A, C and D) contains exon 1, while the other member (E, B, and F) initiates at an in frame AUG start codon at nt 6 of exon 2 (Figure 1A). By using primer sets that distinguish between *KIN-20* isoforms that initiate at the 1^st^ AUG and those that initiate at the 2^nd^ AUG of *KIN-20*, we find that there is ∼3 fold more of the B, E and F isoforms than the A, C, and D isoforms at all time points throughout development (Figure 1B and Figure S1A). By using primer sets that distinguish between the differing 3′ isoforms, we find that there is ∼3 fold more of the B and C isoforms than the A and E isoforms, and ∼10 fold more of the A and E isoforms than the D and F isoforms at all time points throughout development (Figure 1C and Figure S1B). Thus, the B isoform is the major *KIN-20* isoform expressed throughout development (Figure 1 and Figure S1). While the D and F isoforms are only weakly expressed, the A, E and C isoforms are all easily detectable throughout development (Figure 1 and Figure S1). In *Drosophila* Doubletime levels do not cycle (Kloss *et al.* 1998, 2001). By analyzing the expression pattern of all *kin-20* isoforms throughout development, we find that *kin-20* expression is relatively constant throughout development, except for the time prior to and surrounding the L2 molt (Figure 1D). This expression pattern is similar across all *kin-20* isoforms (Figure S1).

The functional significance of these variations in *kin-20* mRNA levels throughout development and between isoforms, as well as the protein expression patterns of *KIN-20* are currently unclear. Despite the uncertainty surrounding these finer points, our experiments reveal that overall *kin-20* is extremely important, as the fertility of *C. elegans* significantly drops ∼2 fold in *kin-20(ok505)* mutant worms relative to WT N2 worms when grown at either 15° or 25° (Figure 1E). As previously shown by RNAi (Banerjee *et al.* 2005), *kin-20(ok505)* mutant worms do not show any defects in the timing of alae formation (Figure S2A), but do exhibit precocious seam cell exit from the cell cycle during the late L4 stage (Figure S2B). In addition, the growth of *C. elegans* is dramatically slowed in *kin-20(ok505)* worms relative to WT N2 as measured by animal size and gonad development at 25° (Figure 1F and Figure S3). This growth delay became particularly apparent during the L3 stage, but may have initiated earlier in development (Figure 1F). In addition, the length of delay increased as the worms continued to develop (Figure 1F and Figure S3).

### *KIN-20* regulates *LIN-42* expression

**Figure 2:**
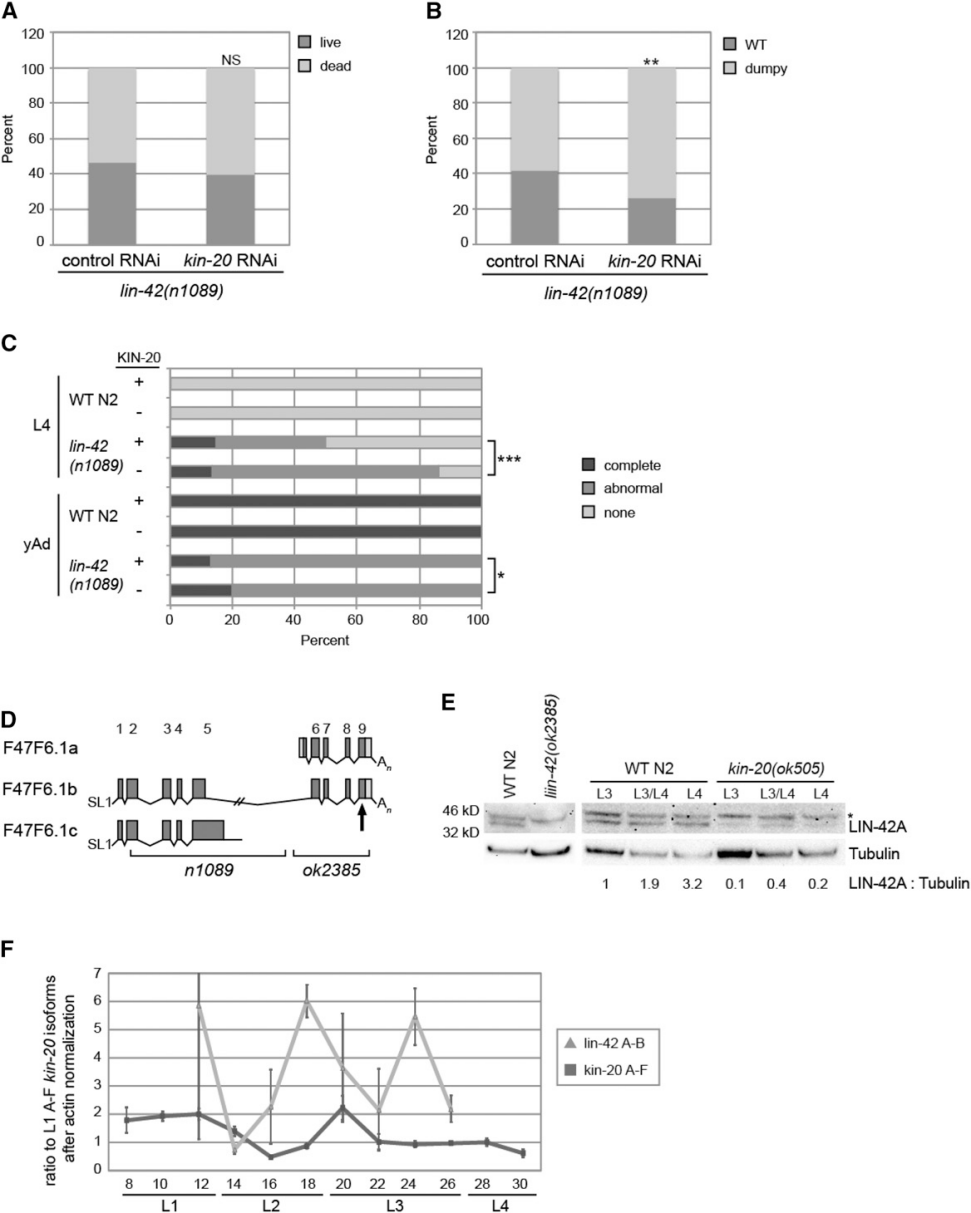
*KIN-20* promotes *LIN-42* expression. (A-C) *lin-42(n1089)* mutant worms were subjected to vector control RNAi or *kin-20* RNAi and analyzed after growth at 25°C. The percentage of dead worms (A) and dumpy worms (B) after growth for 2 days are shown (N > 150), and were analyzed by a chi-square test (***, *P* < 0.0005). NS equals non-significant. (C) The presence of alae was analyzed in late L4 and yAd worms after growth on vector control or *kin-20* RNAi. N > 20. Alae was classified as complete if it extended continuously over all seam cells in three parallel ridges. Alae was classified as abnormal if it was not complete and/or did not contain three perfectly parallel ridges. (D) Depiction of the *lin-42* gene, based on WormBase. The *n1089* and *ok2385* alleles are marked below the gene diagrams. The site targeted by the *LIN-42* antibody is marked with an arrow. (E) Protein was extracted from mixed stage WT N2 and *lin-42(ok2385)* mutant worms, and synchronized WT N2 worms and *kin-20(ok505)* mutant worms and analyzed by western blotting for *LIN-42* and tubulin. Asterisk denotes non-specific bands. A representative blot from 3 independent experiments is shown. The ratio of *LIN-42* A to tubulin after normalization to LIN-42A in WT N2 at the L3 stage is shown. (F) Synchronized WT N2 worms were grown at 25°C and collected every 2 hr as described in Fig. 1 before analysis by qPCR for all *kin-20* isoforms (A-F) or the A and B isoforms of *lin-42*. The ratio to all isoforms of KIN20 at L1 after normalization to actin is shown. Data are shown from 3 independent experiments. Error bars show s.e.m.

The *KIN-20* homologs Doubletime and CK1ε/δ post-translationally regulate Period protein expression via phosphorylation, which marks Period for degradation (Price *et al.* 1998; Crane and Young 2014). *LIN-42* is the Period protein homolog in *C. elegans* (Jeon *et al.* 1999). Thus, we asked if *KIN-20* similarly impacts *lin-42* expression in *C. elegans* by analyzing both *lin-42* phenotypes and expression patterns in the presence and absence of *kin-20*. To do this we attempted to generate worms that were homozygous for both the *lin-42(n1089)* allele and the *kin-20(ok505)* allele. However, progeny of self fertilized *C. elegans* that were homozygous for the *lin-42(n1089)* mutation and heterozygous for the *kin-20(ok505)* mutation (or alternatively homozygous for the *kin-20(ok505)* mutation and heterozygous for the *lin-42(n1089)* mutation) yielded no progeny that were homozygous for both mutations (Table S2). Growth of *lin-42(n1089)* worms in the presence of *kin-20* RNAi caused a slight, but non-significant increase in the proportion of worms that died by the young adult stage as compared to vector control RNAi (Figure 2A). Taken together, these observations suggest that when *LIN-42* levels are reduced in the absence of *KIN-20*, as is seen in the double mutant, but not through RNAi treatment, lethality results.

We also found that *lin-42* mutant phenotypes were enhanced in the absence of *kin-20*. Due to molting defects *lin-42* mutant worms exhibit a dumpy phenotype as adults (Monsalve *et al.* 2011). We find that significantly more *lin-42(n1089)* mutant worms display this dumpy phenotype when subjected to *kin-20* RNAi as compared to vector control RNAi (Figure 2B). As a member of the heterochronic pathway, *lin-42* mutant worms have previously been shown to exhibit a precocious alae phenotype (Abrahante *et al.* 1998; Van Wynsberghe and Pasquinelli 2014). Our analysis of alae formation found that many *lin-42(n1089)* worms exhibited abnormal alae formation, as defined by alae that was either incomplete (did not extend extend continuously over all seam cells) or was not in three perfectly parallel ridges. At the young adult stage, significantly more animals exhibit complete alae in *lin-42(n1089)* worms grown on *kin-20* RNAi compared to vector control RNAi (Figure 2C). In addition, at the L4 stage, though a similar proportion of *lin-42(n1089)* worms have complete alae, significantly more *lin-42(n1089)* worms exhibit precocious alae formation (as seen by the presence of either complete or abnormal alae) when grown on *kin-20* RNAi compared to vector control RNAi (Figure 2C).

*LIN-42* has 3 isoforms (Figure 2D) (Edelman *et al.* 2016). The largest isoform, B, contains sequence found in both the A and C isoforms (Figure 2D). Work in other labs has suggested that the LIN-42A and B isoforms are of greater functional importance than LIN-42C (Tennessen *et al.* 2006; Monsalve *et al.* 2011; Edelman *et al.* 2016). Using an antibody developed against the C terminal region of *LIN-42*, we were able to clearly visualize the LIN-42A isoform (Figure 2E, Figure S4). In the absence of *KIN-20* we found that LIN-42A levels are severely decreased (Figure 2E, Figure S4). To further analyze the effect of *kin-20* on *lin-42* expression, we compared the expression patterns of *lin-42* isoforms A and B to *kin-20* expression throughout all four larval stages in WT N2 worms (Figure 2F). As expected, *lin-42* levels oscillate throughout development (Figure 2F). The significant decrease in *kin-20* mRNA levels found in the L2 stage is followed by an increase in *lin-42* mRNA levels, while the significant increase in *kin-20* mRNA levels found in the early L3 stage is followed by a decrease in *lin-42* mRNA levels (Figure 2F). Altogether, our work provides a causal link for our observations that *kin-20* knockdown enhances *lin-42* mutant phenotypes by further reducing *LIN-42* levels. This suggests that unlike its homologs *KIN-20* promotes *LIN-42* expression.

### *KIN-20* regulates expression of some microRNAs

**Figure 3:**
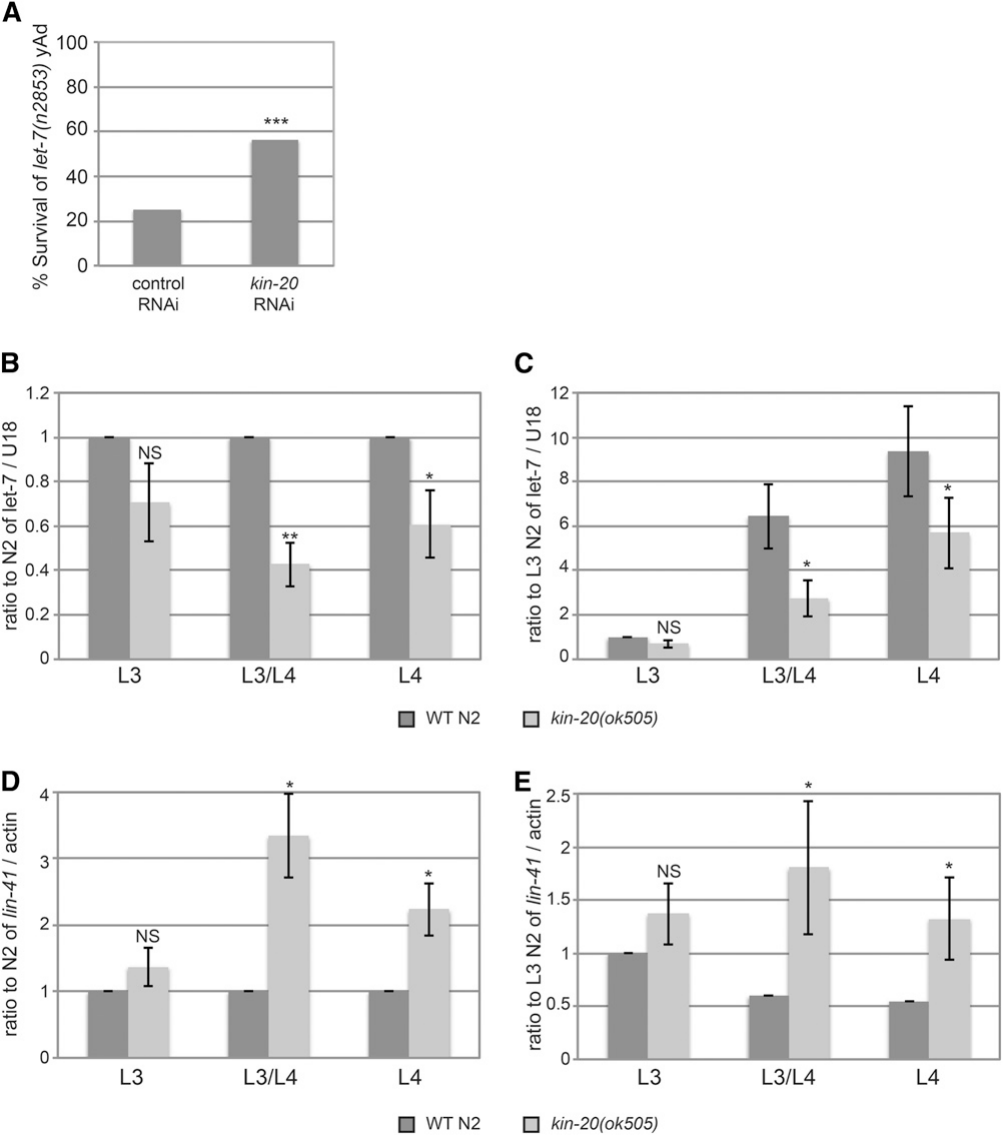
*KIN-20* is important for *let-7* miRNA expression. (A) The percentage of worms alive after growth for 2 days at 25°C on vector control RNAi or *kin-20* RNAi is shown (N > 200), and was analyzed by a chi-square test (***, *P* < 0.0005). (B-E) Synchronized WT N2 or *kin-20(ok505)* mutant worms were grown at 25°C and collected at the L3, L3/L4, or L4 stage based on size and gonad development. RNA was extracted and analyzed by qPCR after reverse transcription for *let-7* (B-C) or *lin-41* (D-E). The average and s.e.m. of RNA levels from at least 6 independent experiments after normalization to U18 mRNA (B-C) or actin mRNA (D-E) are graphed and were analyzed by Student’s *t*-tests (*, *P* < 0.05; **, *P* < 0.005). NS equals non-significant. Levels are shown relative to WT N2 at all time points (B,D) or specifically to WT N2 at the L3 stage (C,E).

We and others have previously shown that *LIN-42* is a transcription factor that acts to inhibit transcription of a multitude of genes including small RNAs called microRNAs (Monsalve *et al.* 2011; Mcculloch and Rougvie 2014; Van Wynsberghe *et al.* 2014). Thus, we hypothesized that *KIN-20* might also regulate microRNAs and other genes through its effects on *LIN-42*. Indeed, Banerjee *et al.* have previously shown that *kin-20* RNAi can rescue the lethal bursting phenotype that occurs in worms with a mutation in the *let-7* microRNA (Banerjee *et al.* 2005). In agreement with these previous findings, we demonstrated that RNAi against *kin-20* significantly rescues the lethal bursting phenotype seen in **let-7*(n2853)* mutant worms after the L4 molt when grown at 25° (Figure 3A), though not as completely as previously reported (Banerjee *et al.* 2005). Since RNAi against *lin-42* or a loss-of-function *lin-42* mutation also rescues the *let-7* bursting phenotype by increasing *let-7* levels (Banerjee *et al.* 2005; Van Wynsberghe *et al.* 2014; Perales *et al.* 2014), we next asked if the ability of *kin-20* RNAi to rescue this *let-7* phenotype was also due to alterations in *let-7* levels. We analyzed mature *let-7* levels at the third larval stage (L3), the L3 molt (L3/L4) and the fourth larval stage (L4) at 25° according to size and gonad development (Figure 1F and Figure S3) in *kin-20(ok505)* and WT N2 worms (Figure 3B-C). We found that *let-7* levels decreased at all time points and that this decrease was significant at the L3 molt and L4 time points (Figure 3B-C). This decrease in *let-7* coincided with a significant ∼2-3 fold increase in mRNA levels of the *let-7* target gene, *lin-41*, in *kin-20(ok505)* mutant worms relative to WT N2 worms (Figure 3D-E).

**Figure 4:**
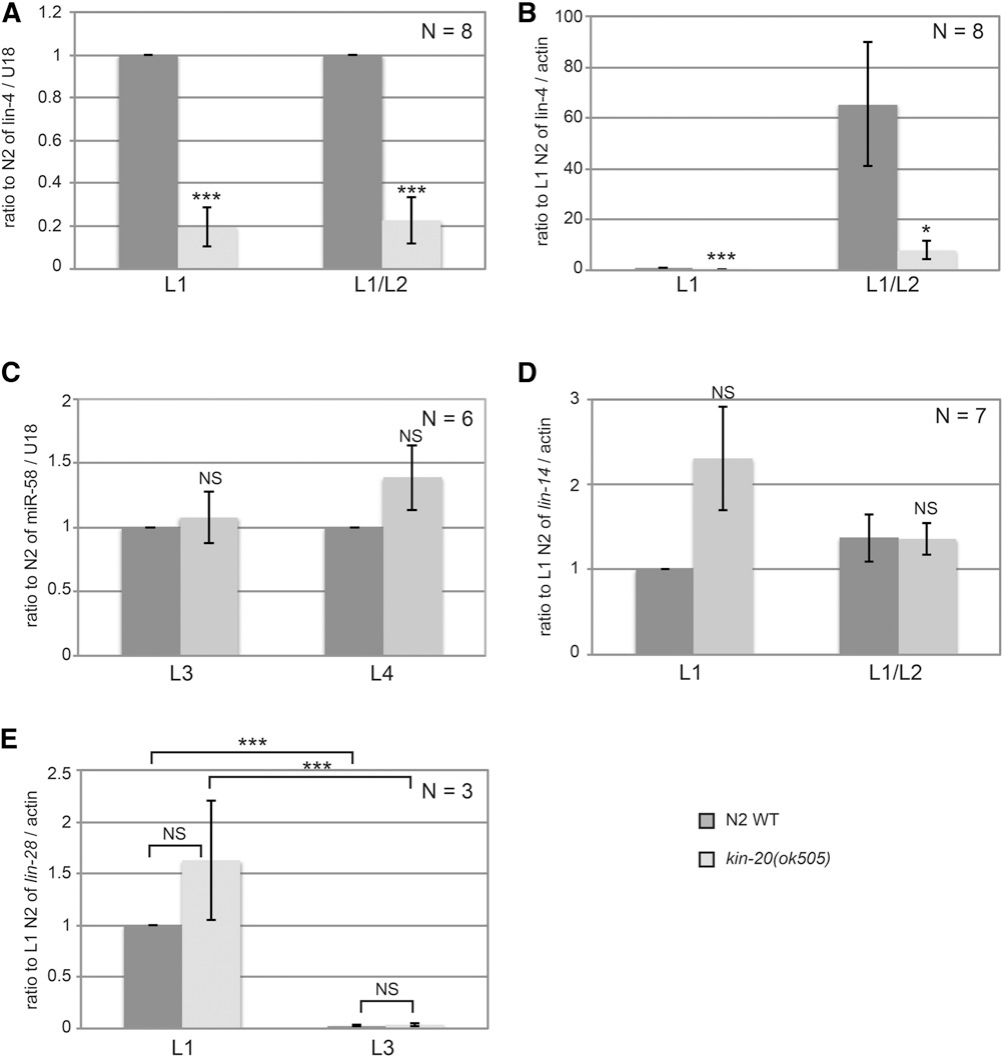
Effect of *KIN-20* on other microRNAs and heterochronic genes. Total RNA was extracted from synchronized WT N2 and *kin-20(ok505)* mutant worms at the time points shown, and analyzed as in Fig. 3 for *lin-4* (A-B), *miR-58.1* (C), or *lin-4* targets *lin-14* (D) and *lin-28* (E). The average and s.e.m. of RNA levels from multiple independent experiments after normalization to U18 (A-C) or actin mRNA (D-E) are shown and were analyzed by Student’s *t*-tests (*, *P* < 0.05; ***, *P* < 0.0005). NS equals non-significant.

Some regulators of miRNA biogenesis act specifically on a single miRNA, while others, like *LIN-42*, act more globally to affect biogenesis of multiple miRNAs (Finnegan and Pasquinelli 2013). To determine if *kin-20* affected other miRNAs, we analyzed the effect of *kin-20* on two other miRNAs: *lin-4* and miR-58.1. *lin-4* is also a member of the heterochronic pathway and is first expressed in the mid-L1 stage (Bracht *et al.* 2010), while miR-58.1 is non-heterochronic gene that is expressed throughout development (Abbott 2011). Like *let-7*, *lin-4* miRNA levels were significantly decreased in *kin-20(ok505)* relative to WT N2 worms (Figure 4A-B). In contrast, levels of miR-58.1 were not altered in *kin-20(ok505)* relative to WT N2 worms at L3 and L4 stages (Figure 4C). *lin-4* functions early in the heterochronic pathway by regulating both *lin-14* and *lin-28* (Ambros 1989). Consistent with a decrease in *lin-4*, we find that *lin-14* mRNA levels are increased early in development in *kin-20(ok505)* relative to WT N2 worms (*P* = 0.076), though there is no effect on *lin-14* mRNA at a later stage (Figure 4D). We also find that *lin-28* mRNA levels are increased, though not significantly, in *kin-20(ok505)* relative to WT N2 worms at the L1 stage (Figure 4E). However, given the importance of *lin-4* in inhibiting *lin-28* mRNA levels (Ambros 1989), and our finding that *lin-4* levels are significantly reduced in *kin-20(ok505)* worms (Figure 1A-B), it is surprising that *lin-28* mRNA levels are significantly decreased in the L3 stage (Figure 4E). This normal expression pattern of *lin-28* mRNA suggests that though *KIN-20* may in part regulate *let-7* via its effects on *lin-4*, *KIN-20* likely predominantly regulates *let-7* downstream of its impacts on *lin-4*.

**Figure 5:**
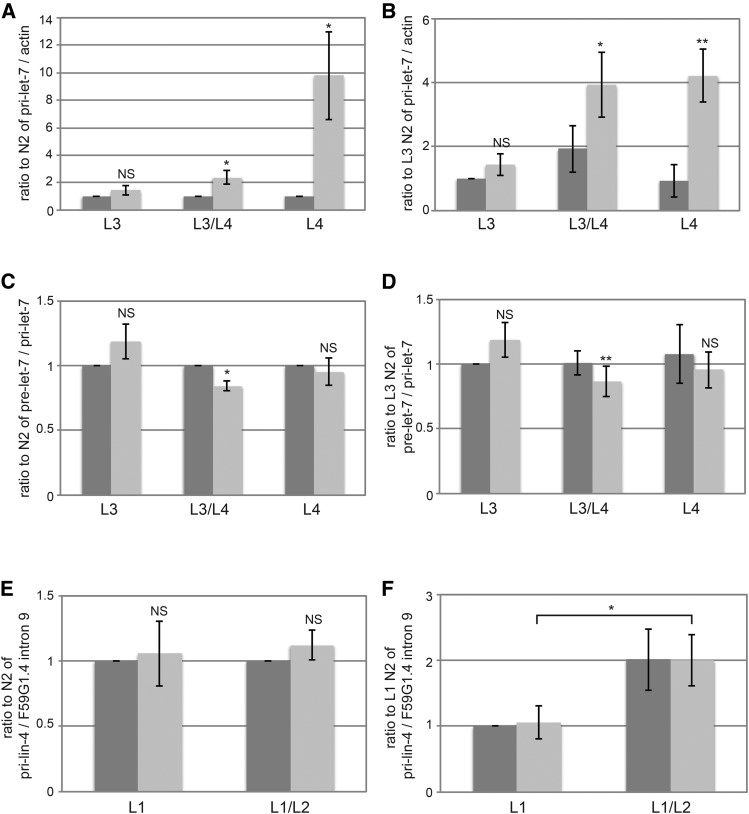
Effect of *KIN-20* on microRNA biogenesis. Total RNA samples from synchronized WT N2 and *kin-20(ok505)* mutant worms that were analyzed for mature *let-7* and mature *lin-4* in Figs. 3 and 4 respectively were also analyzed for pri-let-7 (A-B), pre-let-7 (C-D), and pri-lin-4 (E-F). The average and s.e.m. of RNA levels from 6 (A-D) or 7 (E-F) independent experiments after normalization to actin mRNA (A-B), pri-let-7 levels (C-D), or *F59G1.4* intron 9 are shown and were analyzed by Student’s *t*-tests (*, *P* < 0.05; **, *P* < 0.005). NS equals non-significant. Levels are shown relative to WT N2 at all time points (A,C,E) or specifically to WT N2 at the L3 stage (B,D) or L1 stage (F).

Decreased levels of mature *let-7* could be due to a decrease in transcription of the **let-7** gene, decreased processing of primary or precursor *let-7* RNAs, or decreased stability of mature *let-7*. To distinguish among these possibilities, we analyzed the levels of these RNAs involved in *let-7* biogenesis in the same samples for which mature *let-7* levels had also been analyzed. Transcription initiation at two distinct sites yields two primary *let-7* transcripts that are both subsequently spliced to yield a third primary *let-7* transcript that is ultimately processed into precursor *let-7* (Bracht *et al.* 2004; Van Wynsberghe *et al.* 2011b). Levels of all three primary *let-7* transcripts were analyzed with a single primer set. Primary *let-7* levels were unchanged at the L3 stage, but were significantly increased later in development in *kin-20(ok505)* mutant worms relative to WT (Figure 5A-B). Levels of precursor *let-7* varied slightly at each stage tested in *kin-20(ok505)* mutant worms relative to WT, but overall suggested that pre-*let-7* levels were either minimally altered or that any impacts on pre-*let-7* were negated by its rapid processing into mature *let-7* (Figure 5C-D).

We also analyzed the effect of *KIN-20* on primary *lin-4* in the same samples for which mature *lin-4* had also been analyzed. Primary *lin-4* transcription initiates from two distinct start sites encoded in the 9^th^ intron of the ubiquitously expressed *F59G1.4* gene (Bracht *et al.* 2010). Levels of both primary *lin-4* transcripts were analyzed with a single primer set and normalized to *F59G1.4* intronic sequence. Unlike primary *let-7*, we found that *kin-20* had no impact on primary *lin-4* levels at either time point (Figure 5E-F), suggesting that *KIN-20* acts post-transcriptionally to regulate *lin-4* expression.

**Figure 6:**
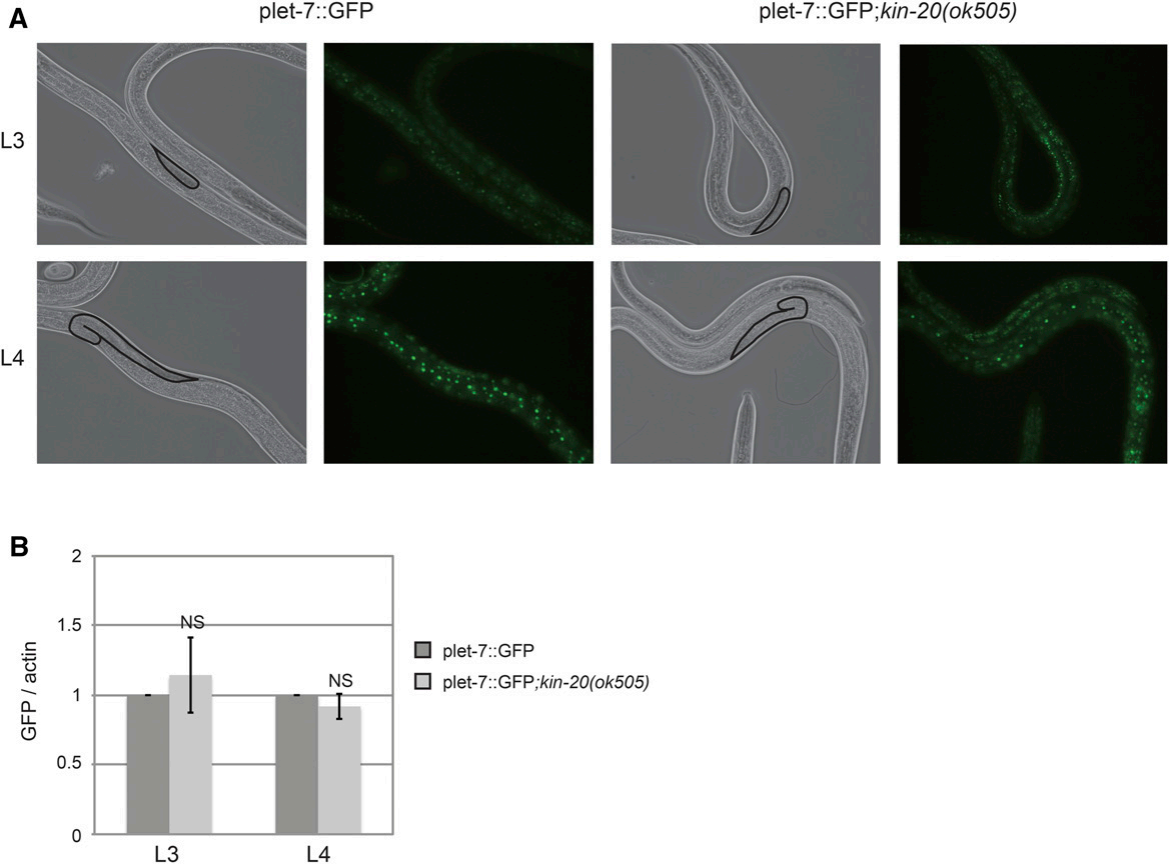
*KIN-20* does not impact *let-7* transcription. (A) Representative images of WT or *kin-20(ok505)* mutant worms expressing the plet-7::GFP reporter at L3 and L4 stages. Fluorescent micrographs were captured under equivalent exposure times. (B) Total RNA was isolated from synchronized WT N2 or *kin-20(ok505)* mutant worms expressing the plet-7::GFP reporter during the L3 and L4 stages. The level of GFP after actin mRNA normalization relative to WT N2 worms was calculated from 3 independent experiments. Error bars show s.e.m. NS equals non-significant.

To assess if *KIN-20* acted as a transcriptional or post-transcriptional regulator of pri-let-7, we utilized an integrated reporter that expresses GFP from ∼1568 bp of *let-7* promoter sequence (Kai *et al.* 2013), and visualized *let-7* expression in the seam cells. As expected (Kai *et al.* 2012), *let-7* promoter driven expression of GFP was stronger at the L4 stage compared to the L3 stage (Figure 6A). However, there was no consistent difference in GFP expression in the presence *vs.* absence of *kin-20* as visualized by fluorescent microscopy (Figure 6A). Because this is a stable GFP reporter, we also analyzed GFP mRNA levels to better understand the impact of *kin-20* on *let-7* transcription. Quantitative real time PCR analysis showed that GFP mRNA levels were unchanged in *kin-20(ok505)* mutant worms relative to WT N2 worms at both the L3 and L4 stages (Figure 6B). Altogether these results suggest that in the absence of *kin-20* a blockage of primary *let-7* processing causes an increase in pri-let-7 levels coincident with a decrease in mature let-7.

### *KIN-20* mainly regulates *let-7* independently of its effects on *LIN-42*

**Figure 7:**
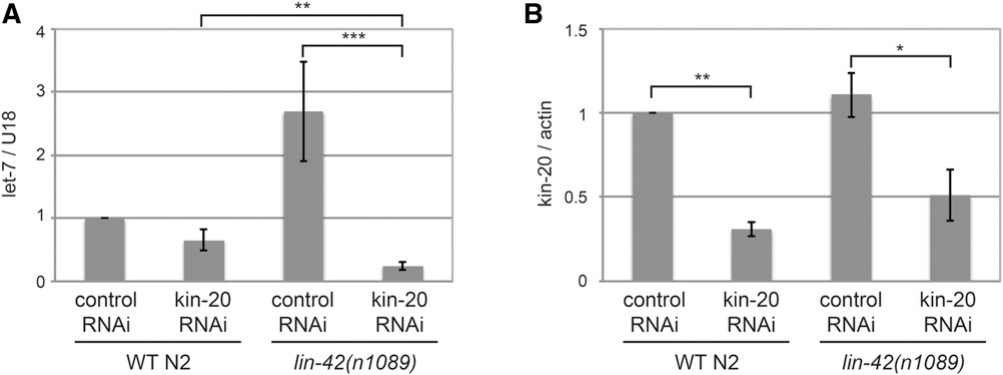
*KIN-20* mainly impacts miRNA levels independently of *LIN-42*. Total RNA was extracted from synchronized WT N2 or *lin-42(n1089)* mutant worms subjected to 2-generation vector control RNAi or *kin-20* RNAi and collected at the L3/L4 stage. RNA was analyzed by qPCR after reverse transcription. The average and s.e.m. of RNA levels from 3 independent experiments are shown and were analyzed by Student’s *t*-tests (*, *P* < 0.05; **, *P* < 0.005; ***, *P* < 0.0005). (A) Levels of *let-7* miRNA after normalization to U18 mRNA relative to WT N2 subjected to vector control RNAi. (B) Levels of *kin-20* mRNA after actin mRNA normalization relative to WT N2 subjected to vector control RNAi.

To further test if *KIN-20* affected *let-7* miRNA expression by regulating *LIN-42* protein levels, we analyzed the levels of *let-7* miRNA in WT N2 or *lin-42(n1089)* worms grown in the presence of vector control or *kin-20* RNAi (Figure 7). RNAi against *kin-20* did not cause the growth delay seen in *kin-20(ok505)* mutant worms (Figure S5). As expected, *let-7* levels of worms grown on vector control RNAi were ∼2.5 fold increased in *lin-42(n1089)* mutant worms relative to WT N2 worms (Figure 7A) (Van Wynsberghe *et al.* 2014). Levels of *let-7* were only slightly decreased in WT N2 worms treated with *kin-20* RNAi (Figure 7A). This smaller effect, as compared to the change in *let-7* levels in a *kin-20* mutant (Figure 3), was likely due to the only ∼2-3 fold decrease in *kin-20* levels from *kin-20* RNAi treatment (Figure 7B). Despite this, knockdown of *kin-20* levels by RNAi significantly reduced *let-7* levels by 10 fold in *lin-42(n1089)* mutant worms (Figure 7A). Levels of *let-7* were also significantly decreased by ∼3 fold in *lin-42(n1089)* mutant worms treated with *kin-20* RNAi compared to WT N2 worms treated with *kin-20* RNAi (Figure 7A). Because *let-7* levels are significantly different in the absence of both *lin-42* and *kin-20* compared to just the absence of *lin-42* or *kin-20*, this together with our other data suggests that *KIN-20* predominantly acts independently of its effects on *LIN-42* to regulate *let-7* levels.

## Discussion

Here we further characterize the Period protein kinase homolog *KIN-20* and demonstrate that *KIN-20* regulates both the Period protein homolog **LIN-42** and specific miRNAs like *let-7* and *lin-4*. We show that though all *KIN-20* isoforms are expressed throughout development, there is great variation in the levels of expression of these isoforms and that the B isoform of *kin-20* is the most expressed (Figure 1 and Figure S1). We find that *kin-20* mutant worms have decreased progeny numbers, grow slowly and exhibit aberrant seam cell development, but not alae production (Figure 1 and Supplementary Figures 2 and 3). We show that LIN-42A levels are decreased and *lin-42* mutant phenotypes are enhanced when *KIN-20* levels are decreased (Figure 2). In addition, in the absence of *KIN-20*, mature *let-7* levels decrease concordantly with an increase in primary-*let-7* levels (Figures 3 and 5). *KIN-20* similarly affects mature *lin-4* levels, but not mature miR-58.1 levels, and pri-*lin-4* levels are not altered in *kin-20* mutant worms (Figures 4 and 5). *KIN-20* mediates these effects on *let-7* and *lin-4* post-transcriptionally because GFP mRNA and protein levels, when placed under the control of the *let-7* promoter, are not altered in the absence of *KIN-20* (Figure 6). *KIN-20* impacts *LIN-42* and *let-7*, and *LIN-42* has been previously shown to inhibit *let-7* expression (Mcculloch and Rougvie 2014; Van Wynsberghe *et al.* 2014; Perales *et al.* 2014). However, because *let-7* levels significantly differ in *lin-42* mutant worms that express decreased levels of *kin-20*, we can conclude that *KIN-20* mainly regulates *let-7* independently of *LIN-42*. These results uncover a new mechanism used to control both the conserved Period protein homolog *LIN-42* and the important, conserved microRNA *let-7*, and thus developmental and rhythmic processes.

Developmental timing in *C. elegans* is maintained by the heterochronic pathway, which is comprised of a complex network of genes (Rougvie and Moss 2013). When absent, heterochronic genes either cause precocious developmental phenotypes or reiteration of, and thus retarded, developmental phenotypes. Gain-of-function mutations in heterochronic genes cause the opposite developmental effect. Measurement of developmental delays in *C. elegans* is most commonly done through analysis of hypodermal seam cells, which exhibit regular division patterns throughout larval growth before fusing and secreting alae, a series of cuticular ridges, in the young adult stage (Resnick *et al.* 2010). Consistent with previously reported results (Banerjee *et al.* 2005), we find that *kin-20(ok505)* mutant worms precociously exit the cell cycle at late L4, represented by a decrease in the number of hypodermal seam cell nuclei at this stage (Figure S2B). Additionally, *kin-20(ok505)* mutant worms exhibit wild type timing of alae formation (Figure S2A) (Banerjee *et al.* 2005). However, *kin-20* RNAi enhances the precocious alae phenotype in *lin-42(n1089)* mutant worms (Figure 2C). In addition, *kin-20(ok505)* mutants exhibit altered expression of multiple heterochronic genes including *lin-42*, **let-7** and **lin-4** (Figures 2-4). Despite these impacts on gene expression, the finding that *KIN-20* itself does not exhibit aberrant alae development, and therefore the altered developmental timing typical of heterochronic genes, suggests that *KIN-20* is not a traditional heterochronic gene.

Like other Period proteins, expression of *LIN-42*, the *C. elegans* Period protein homolog, oscillates throughout development (Jeon *et al.* 1999; Tennessen *et al.* 2006; Monsalve *et al.* 2011; Edelman *et al.* 2016). Given the role of Doubletime in regulating Period, we hypothesized that the Doubletime homolog *KIN-20* would similarly impact *LIN-42*. However, we found that levels of the LIN-42A isoform were decreased and that multiple *lin-42* mutant phenotypes were enhanced in *kin-20(ok505)* mutant worms (Figure 2). Since LIN-42A levels were decreased in *kin-20(ok505)*, we might expect *kin-20(ok505)* worms to phenocopy *lin-42(ok2385)* worms, which delete LIN-42A and contain a large deletion in the C-terminal region of LIN-42B (Edelman *et al.* 2016). Like *lin-42(ok2385)*, *kin-20(ok505)* worms have a significantly reduced brood size, growth delays, and precocious alae (Figures 1 and 2) (Edelman *et al.* 2016). Similarly, *lin-42(n1089)* mutant worms, which contain a large deletion at the N terminus of *lin-42*, when grown in the absence of *kin-20* might be expected to phenocopy *lin-42(ox461)* worms, which lack all *lin-42* isoforms (Edelman *et al.* 2016). Unfortunately, we were unable to analyze the phenotypes of *lin-42(n1089);kin-20(ok505)* due to their lethality (Table S2). However, we did analyze the phenotypes of *lin-42(n1089)* worms subjected to *kin-20* RNAi. Despite *kin-20* RNAi only decreasing *kin-20* levels ∼2-3 fold (Figure 7B), we found that *lin-42* mutant phenotypes were enhanced after *kin-20* RNAi treatment (Figure 2). *lin-42(ox461)* worms exhibited enhanced larval arrest and growth delays (Edelman *et al.* 2016). In contrast, *lin-42(n1089)* worms subjected to *kin-20* RNAi did not show any significant growth delays (Figure S5). Though the proportion of worms exhibiting some type of precocious alae (partial or complete) did not change in *lin-42(ox461)* worms, more *lin-42(ox461)* worms exhibited complete alae (Edelman *et al.* 2016). We also found synergistic effects of *lin-42* and *kin-20* on alae production (Figure 2C). However, we found that the proportion of worms exhibiting some type of precocious alae (abnormal or complete), but not the number of worms exhibiting complete alae, significantly increased in *lin-42(n1089)* worms subjected to *kin-20* RNAi (Figure 2C). Thus, our results suggest that both *lin-42* and *kin-20* act in similar pathways, in addition to their distinct, crucial functions in development. Altogether, these results suggest that instead of destabilizing *LIN-42*, *KIN-20* acts to stabilize or promote LIN-42A expression.

There are three *LIN-42* isoforms that have each been shown to be important for proper developmental timing (Edelman *et al.* 2016). However, there is still much to be determined about whether the isoforms have distinct functions and/or expression patterns. LIN-42C and the N terminal region of LIN-42B contain the conserved protein interaction (PAS) domain characteristic of Period proteins, while LIN-42A and the C terminal region of LIN-42B contain the conserved nuclear localization signal and the SYQ and LT regions which contain multiple ser, tyr and gln or leu and thr amino acids respectively (Figure 2D) (Jeon *et al.* 1999; Tennessen *et al.* 2006). LIN-42A and the C terminal region of LIN-42B are thought to contain the most important regions of *LIN-42* since mutations in LIN-42C and the N terminus of LIN-42B can be rescued by overexpression of LIN-42A (Tennessen *et al.* 2006). In contrast, overexpression of LIN-42C does not rescue mutations in LIN-42A or the C terminus of LIN-42B (Tennessen *et al.* 2006). In addition, LIN-42A is particularly important for regulating molting and seam cell development in *C. elegans* (Monsalve *et al.* 2011). Unfortunately, our western blotting analysis only enabled detection of the LIN-42A isoform. Thus it is still unclear if and how *KIN-20* regulates the other isoforms of *LIN-42*. To start to address this issue we compared levels of *lin-42* A and B mRNA to levels of all *kin-20* isoforms by qRT-PCR (Figure 2F). In *Drosophila*, Period engages in an autoregulatory negative feedback loop to inhibit transcription of the Period gene (Peschel and Helfrich-Förster 2011). Thus a decrease in *kin-20* levels, as seen in the L2 stage (Figure 2F), would cause a decrease in LIN-42A levels followed by a subsequent increase in *lin-42* mRNA levels. Similarly, an increase in *kin-20* levels, as seen at the beginning of L3 (Figure 2F), would cause an increase in LIN-42A levels, and thus a subsequent decrease in *lin-42* mRNA. Though both of these associations do occur (Figure 2F), more studies are needed to clearly show the impact of *KIN-20* on the expression of individual *LIN-42* isoforms. In addition, it is unclear if such an autoregulatory negative feedback loop even exists in *C. elegans*, since previous work in the Rougvie lab has shown that *lin-42* levels still oscillate in the absence of functional *LIN-42* protein (Jeon *et al.* 1999). Regardless, because we find that *lin-42* mutant phenotypes are enhanced in *kin-20(ok505)* mutant worms, it is most likely that if *KIN-20* regulates the other *LIN-42* isoforms it does so in a similar manner to its effects on LIN-42A. Based on its homology with Doubletime, it is also most likely that *KIN-20* mediates these effects via phosphorylation.

*LIN-42* has previously been shown to act as a transcription factor that negatively regulates the expression of numerous target genes including the miRNAs *let-7*, *lin-4* and miR-58.1 (Mcculloch and Rougvie 2014; Van Wynsberghe *et al.* 2014; Perales *et al.* 2014). Thus, because of its impact on *LIN-42*, we hypothesized that *KIN-20* might also regulate these miRNAs. This hypothesis was further supported by the finding that *kin-20* RNAi rescued the lethal **let-7*(n2853)* bursting phenotype (Figure 3A) (Banerjee *et al.* 2005), though we found that the degree of rescue was less than previously reported (Banerjee *et al.* 2005). The *n2853* allele is a temperature-sensitive point mutation in the seed sequence of the mature miRNA that decreases the levels of mature *let-7* by more than 5 fold relative to WT (Reinhart *et al.* 2000; Bagga *et al.* 2005; Chatterjee and Großhans 2009; Zisoulis *et al.* 2012). Reduced *let-7* levels then cause bursting through the vulva after the L4 molt at 25° (Reinhart *et al.* 2000). When their levels are reduced, various members of the heterochronic pathway and other pathways have been found to rescue this lethal phenotype. For example, *lin-42* RNAi rescues the lethal **let-7*(n2853)* bursting phenotype by causing an ∼2.5 fold increase in *let-7* levels (Banerjee *et al.* 2005; Van Wynsberghe *et al.* 2014). Surprisingly, we found that instead of expressing increased *let-7* levels, *kin-20(ok505)* mutant worms showed ∼2 fold decreased levels of *let-7* relative to WT (Figure 3B-C) and a corresponding increase in the *let-7* target *lin-41* (Figure 3D-E). This further reduction in *let-7* levels would be expected to increase **let-7*(n2853)* lethality, not reduce it as we found (Figure 3A). Thus, our results suggest that *KIN-20* suppresses the *let-7* bursting phenotype in a manner that is independent of the heterochronic pathway. For example, *KIN-20* may impact the expression of other genes involved in vulva formation in order to rescue this lethal phenotype.

*KIN-20* impacts miRNA expression differently from *LIN-42* since levels of the *lin-4* miRNA, but not the constitutively expressed miR-58.1 miRNA were decreased in *kin-20(ok505)* mutant worms (Figure 4A-C). *lin-4* acts early in the heterochronic pathway to regulate developmental timing (Ambros 1989). Surprisingly, levels of the *lin-4* targets *lin-14* and *lin-28* were only increased initially in *kin-20(ok505)* mutant worms (Figure 4D-E). *LIN-28* normally acts to downregulate expression of mature *let-7*, and *let-7* levels only increase during the L3 stage after *LIN-28* expression decreases as a result of *lin-4* expression (Resnick *et al.* 2010; Lee *et al.* 2016). Thus, it is possible that *KIN-20* could regulate *let-7* indirectly via *lin-4* and *lin-28*. However, the finding that *lin-28* mRNA levels decrease normally in the L3 stage suggests that if *KIN-20* regulates *let-7* via *lin-4* and *lin-28*, *KIN-20* must also regulate *let-7* downstream of its effects on *lin-4* (Figure 4E).

Unlike *LIN-42*, we find that *KIN-20* acts post-transcriptionally to regulate levels of mature *let-7* and *lin-4*. *KIN-20* had no effect on primary *lin-4* levels (Figure 5E-F). Though primary *let-7* levels were increased in *kin-20(ok505)* mutant worms (Figure 5A-B), transcription from the *let-7* promoter was not affected since GFP mRNA levels were unchanged in *kin-20(ok505)* mutant worms (Figure 6). The increase in primary *let-7* levels concordant with the decrease in mature *let-7* levels suggests that *KIN-20* does not impact mature *let-7* stability. In addition, the fact that precursor *let-7* levels remain mostly unchanged (Figure 5C-D) suggests that *KIN-20* most likely regulates the processing of primary *let-7* into precursor *let-7*. There are many proteins that regulate mature *let-7* production at all steps in miRNA biogenesis (Finnegan and Pasquinelli 2013; Lee *et al.* 2016). Although the exact mechanism that *KIN-20* utilizes to have this effect is still unclear, *KIN-20* likely impacts another *let-7* regulator through phosphorylation.

Our results suggest that *KIN-20* positively regulates both *LIN-42* and *let-7*. Previous work in our lab and others has shown that *LIN-42* also negatively regulates *let-7* (Mcculloch and Rougvie 2014; Van Wynsberghe *et al.* 2014; Perales *et al.* 2014). Thus, though it is possible that *KIN-20* positively regulates LIN-42A to positively regulate *let-7* expression, this model does not fit with previously published reports that *LIN-42* acts as an inhibitor of miRNA transcription (Mcculloch and Rougvie 2014; Van Wynsberghe *et al.* 2014; Perales *et al.* 2014). Though we cannot rule out that *KIN-20* regulates *let-7* through its effects on LIN-42A, our data suggests that instead *KIN-20* mainly regulates *let-7* independently of its effects on *LIN-42*. First, LIN-42A levels decrease in the absence of *kin-20* (Figure 2). Because *lin-42(n1089)* specifically eliminates LIN-42B and C isoforms, reducing *kin-20* levels by RNAi in *lin-42(n1089)* worms will reduce LIN-42A levels and thus should enhance *lin-42* knock-out phenotypes. Accordingly, we find that *lin-42(n1089)* worms subjected to *kin-20* RNAi do indeed exhibit enhanced *lin-42* mutant phenotypes (Figure 2). Since *LIN-42* represses *let-7* expression (Mcculloch and Rougvie 2014; Van Wynsberghe *et al.* 2014; Perales *et al.* 2014), and we have found that LIN-42A levels decrease in the absence of *kin-20* (Figure 2), we would expect *let-7* levels to increase in a *kin-20* mutant that expresses decreased LIN-42A levels. However, the opposite occurs, suggesting that *kin-20* must impact *let-7* expression independently of its affects on *LIN-42* (Figure 3). Second, *LIN-42* regulates ∼30% of all miRNAs at the L4 stage (Van Wynsberghe *et al.* 2014; Perales *et al.* 2014). Thus, if *KIN-20* acted predominantly through *LIN-42*, we would expect *KIN-20* to similarly affect multiple miRNAs. Instead, we find that *KIN-20* does not affect miR-58.1 (Figure 5), which is regulated by *LIN-42* (Van Wynsberghe *et al.* 2014). In addition, since *LIN-42* transcriptionally regulates *let-7* (Mcculloch and Rougvie 2014; Van Wynsberghe *et al.* 2014; Perales *et al.* 2014), we would expect *KIN-20* to also act at the transcriptional level if it mainly impacted *let-7* expression via *LIN-42*. However, our results suggest that *KIN-20* regulates *let-7* post-transcriptionally (Figure 6). To further test if *KIN-20* regulates *let-7* via *LIN-42*, we analyzed *let-7* levels in *lin-42(n1089)* mutant worms treated with *kin-20* RNAi. If *KIN-20* acted solely via *LIN-42*, we would expect *let-7* levels to be the same in *lin-42(n1089)* worms treated with vector control or *kin-20* RNAi. Instead we find that *let-7* levels are significantly reduced in *lin-42(n1089)* worms treated with *kin-20* RNAi compared to either *lin-42(n1089)* worms treated with vector control RNAi or WT N2 worms treated with *kin-20* RNAi (Figure 7). Thus the decrease in both *lin-42* and *kin-20* levels acts to enhance the reduction in *let-7* levels. In summary, though these results do not exclude the possibility that the decrease in *let-7* levels in *kin-20* mutant worms is dependent on LIN-42A, these results strongly support another mechanism, that is independent of LIN-42A, by which *kin-20* regulates *let-7* levels.

Altogether these results identify *KIN-20* as a new, important regulator of *LIN-42* and the conserved *lin-4* and *let-7* miRNAs. These results also highlight several important differences between *KIN-20* and its homologs Doubletime and CKIε/δ, and increase our understanding of how rhythmic and developmental processes are regulated in *C. elegans*.
